# Supplementary material for: “Not All Who Wander Are Lost”: The Life Transitions and Associated Welfare of Pack Mules Walking the Trails in the Mountainous Gorkha Region, Nepal
Source: Animals (Basel). 2022 Nov 15;12(22):3152. doi: 10.3390/ani12223152 (PMC9686551; doi:10.3390/ani12223152)
Supplement: Supplementary file 1 [file animals-12-03152-s001.zip › Supplementary material-Table S1.pdf]

Supplementary material

Livelihoods Survey

Livelihoods survey questions
village/area name
GPS
What is your age?
Gender
What is your current job role?
Who is the head of your household?
How many adults (everyone 18 and over) are in your household (i.e both permanent and temporary homes)?
How many adults does your work here support?
How many male children (under 18) are in your household?
How many female children (under 18) are in your household?
What is your ethnic group?
What is your religion?
Do you own your permanent home, rent it, or stay with extended family?
Do you own or rent any land in your permanent home?
Do you have any of the following in your household?
How much does your household earn from work involving your equids per week (in total, including money saved and spent)?
How much does your household earn per week (in total, including money saved and spent)?
What percentage of your annual income is dependent on your equids?
Do you have any other sources of income?
Does your household own any equids?
How many equids does your household own?
How many handlers do you employ?
What is the average age of your equids?
On average, how many years do you keep the same equids for?
On average, how much did you pay for an equid?
What is your education level?

How many years have you worked with equids for?

Did you have any previous experience of working with equids?

Did your family work with equids when you were child?
