# Supplementary material for: “Not All Who Wander Are Lost”: The Life Transitions and Associated Welfare of Pack Mules Walking the Trails in the Mountainous Gorkha Region, Nepal
Source: Animals (Basel). 2022 Nov 15;12(22):3152. doi: 10.3390/ani12223152 (PMC9686551; doi:10.3390/ani12223152)
Supplement: Supplementary file 1 [file animals-12-03152-s001.zip › Supplementary material-Table S2.pdf]

Supplementary Material

EARS scoping protocol sections

Question	Options
Please indicate the number of equids present at location	
Type of location	
	Farm
	Community (hamlet, village, town etc)
	Control post
	Assembly centre
	Market
	Slaughter house
	Stud
	Dairy
	Pharmaceutical
	Brick kiln
	Therapy centre
	Tourism centre/ tourist spot
	Festival
	Guardian home
	Other (please specify)
	Unknown (I don’t know the answer)
	Not assessed or not available
Name of the location	
Please collect GPS coordinates	
Assessor name	
Data inputter	
Participant ID	
Is it possible to assess the housing conditions of the equid?	No
	Yes
	Unknown (don’t know the answer)

	Not assessed or not available
Presence of water point?	No
	Yes, trough
	Yes, automatic drinker
	Yes, natural source
	Unknown (I don’t know the answer)
	Not assessed or not available
Water access (outside the working period)?	Free access
	Limited access
	No access
	Unknown (I don’t know the answer)
	Not assessed or not available
Quality of the water provided?	Clean
	Partially dirty
	Dirty
	Unknown (I don’t know the answer)
	Not assessed or not available
Please indicate how the equid is kept during assessment	Loose
	Restrained
	Bridle or similar
	Full harness/ work equipment
	Harness and hitched to a vehicle
	Full harness but nothing on the head
	Other (please specify)
	Unknown (I don’t know the answer)
	Not assessed or not available
Species	Donkey
	Hybrid
	Horse
General attitude of the equid at a distance	At ease-relaxed, calm and/or resting
	Alert and actively interested in surroundings
	Agitated, aggressive, hyper-reactive/hyper-vigilant
	Apathetic, depressed, withdrawn

	Unknown (I don’t know the answer)
	Not assessed or not available
Is the owner in direct contact with the equid?	No
	Yes
	Unknown (I don’t know the answer)
	Not assessed or not available
Is the equid with other animals?	No
	Yes, physical contact
	Yes, visual contact
	Unknown (I don’t know the answer)
	Not assessed or not available
What species are present?	Donkey
	Hybrid
	Horse
	Ruminants
	Small ruminants
	Camelids
	Other (Please specify)
	Unknown (I don’t know the answer)
	Not assessed or not available
Response to observer	Does not move
	Friendly approach (turning head towards observer)
	Moves head away from observer
	Moves whole body away when observer approaches
	Aggressive behaviour towards the observer –flattens ears, attempts to bite or kick
	Unknown (I don’t know the answer)
	Not assessed or not available
Please indicate signs of fear and distress	No signs of fear and distress present
	Aggressive behaviour
	Unpredictable or sudden movements
	Showing the whites of the eyes
	Sudden startle responses when standing quietly (Rapidly lifting the head)
	Trembling

	Head shyness
	Completely withdrawn/ shut down
	Other (please specify)
	Unknown (I don’t know the answer)
	Not assessed or not available
Sex/ gender	Mare
	Gelding
	Jack/stallion/ entire
	Unknown (I don’t know the answer)
	Not assessed or not available
Age category	Not assessed
	Less than or equal to 1 year old (foal)
	More than 1, less than or equal to 3 years old (adolescent)
	More than 3, less than or equal to 5 years old (young adult)
	More than 5, less than or equal to 5 years old (adult)
	More than 20 years old (geriatric)
	Unknown (I don’t know the answer)
	Not assessed or not available
Please indicate the size of the equid	Miniature
	Pony
	Full size/ horse
	Unknown (I don’t know the answer)
	Not assessed or not available
Please indicate the type of equid	Working equid
	Production equid
	Equid in entertainment
	Feral equid
	Pet equid
	Field companion
	Equid in a sanctuary
	Sport/ competition equid
	Equid used for therapy
	Other (please specify)

	Unknown (I don’t know the answer)
	Not assessed or not available
Other additional information, please specify	
Presence of signs of harmful practices that may affect welfare	No
	Yes, presence of signs of hot brand
	Yes, presence of signs of firing
	Yes, presence of signs of limb tethering or hobbling
	Yes, presence of signs of amputations or mutilations (ears, tails, nostrils)
	Yes, presence of signs of use of live serrata or similar (e.g. metallic chain in the noseband)/ muzzle
	Yes, other situation (please specify)
	Unknown (I don’t know the answer)
	Not assessed or not available
Other/ additional information, please specify	
Body condition?	Very thin/poor
	Thin/moderate
	Ideal
	Fat
	Very fat/ obese
	Unknown (I don’t know the answer)
	Not assessed or not available
Please indicate which feed types are routinely fed	Hay
	Haylage
	Silage
	Mix/muesli
	Cereal grains
	Legumes/ pulses
	Chopped fibre/ chaff
	Other prepared fibres
	Grass
	Browse
	Straw/stover
	Milk

	Milk pellets
	Creep feed
	Vitamins/ mineral
	Sugar based products
	Other (please specify)
	Unknown (I don’t know the answer)
	Not assessed or not available
For how long is fibre available?	18 to 24 hrs per day
	14 to 18 hrs per day
	Below 14 hrs per day
	Unknown (I don’t know the answer)
	Not assessed or not available
Are there any signs of skin alterations?	No
	Yes, open wounds
	Yes, scars
	Yes, alopecia
	Yes, sarcoids
	Yes, swellings
	Other (please specify)
	Unknown (I don’t know the answer)
	Not assessed or not available
Is the equid lame?	Not lame
	Yes (severely lame/ unable to walk/ laid down/ unable to raise)
	Yes (lame but still moving)
	Unknown (I don’t know the answer)
	Not assessed or not available
Does the equid have any signs of hoof neglect or disease?	No
	Yes
	Unknown (I don’t’ know the answer)
	Not assessed or not available
Are limbs aligned correctly?	No
	Yes
	Unknown (I don’t know the answer)

	Not assessed or not available
Please indicate obvious signs of illness (other than conditions previously recorded)	No signs present
	Nasal discharge
	Eye discharge
	Signs of diarrhoea
	Unhealthy coat
	Significant discharge from the penis or vulva
	Abdominal pain
	Other (specify)
	Unknown (I don’t know the answer)
	Not assessed or not available
Is the equid’s coat healthy?	Healthy
	Unhealthy
	Unknown (I don’t know the answer)
	Not assessed or not available
Please indicate apparent general health status of the equid	Good
	Fair
	Poor
	Unknown (I don’t know the answer)
	Not assessed or not available
Main problems affecting the equid, according to the owners?	
Any general additional information?	
