# Supplementary material for: “Not All Who Wander Are Lost”: The Life Transitions and Associated Welfare of Pack Mules Walking the Trails in the Mountainous Gorkha Region, Nepal
Source: Animals (Basel). 2022 Nov 15;12(22):3152. doi: 10.3390/ani12223152 (PMC9686551; doi:10.3390/ani12223152)
Supplement: Supplementary file 1 [file animals-12-03152-s001.zip › Supplementary material-Table S3.pdf]

Supplementary material

Semi-structured interview core questions relevant to this study

SSI questions
How many mules do you own / work with?
How many handlers work for you? How many mules are they each responsible for?
How many mules are they each responsible for?
How long have you owned / worked with mules?
Why did you start owning / working with mules?
What did you do before you owned / worked with mules to make an income?
What impact would it have on your family if you didn't own / work with mules?
What are your mules currently used for?
If the mules are used to transport goods, what type of goods do they transport?
What are the main risks to the mules during these journeys?
Why are mules used for this job (i.e. what are the alternatives?)
What role do mules have in your community?
Where do you get your mules from?
Are they already trained for work before you buy them?
Do you know what work they were previously doing?
How do you train your mules for work here?
What age do you purchase them?
How much do they cost?
How long do you keep each mule? or how long have you owned the mule/s you have now?
What are the main risks to mules on the routes?
What do you do to reduce these risks?
